# Supplementary figures and images for: Identification, expression and variation of the GNPDA2 gene, and its association with body weight and fatness traits in chicken
Source: PeerJ. 2016 Jun 15;4:e2129. doi: 10.7717/peerj.2129 (PMC4911950; doi:10.7717/peerj.2129)

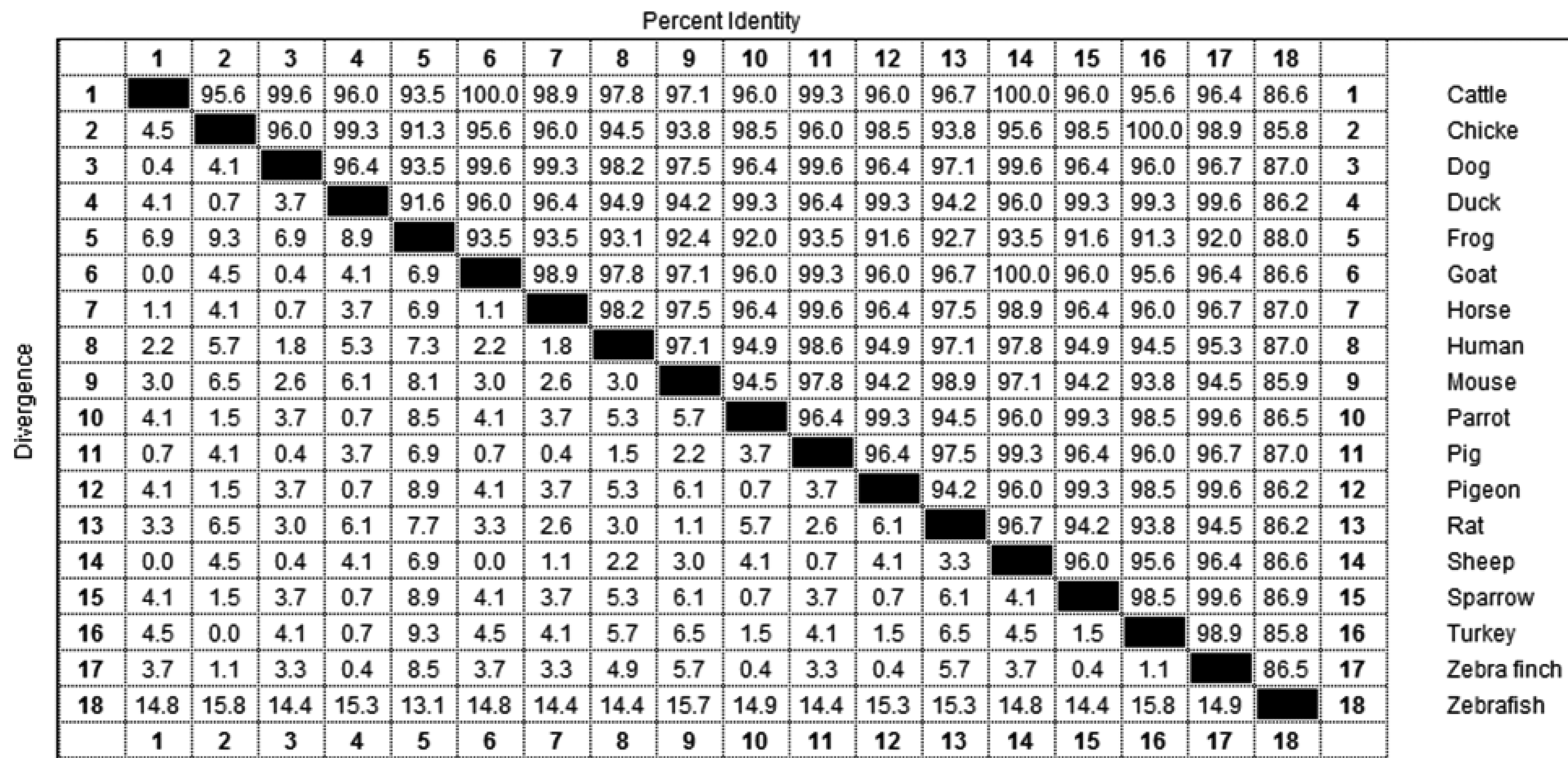

A

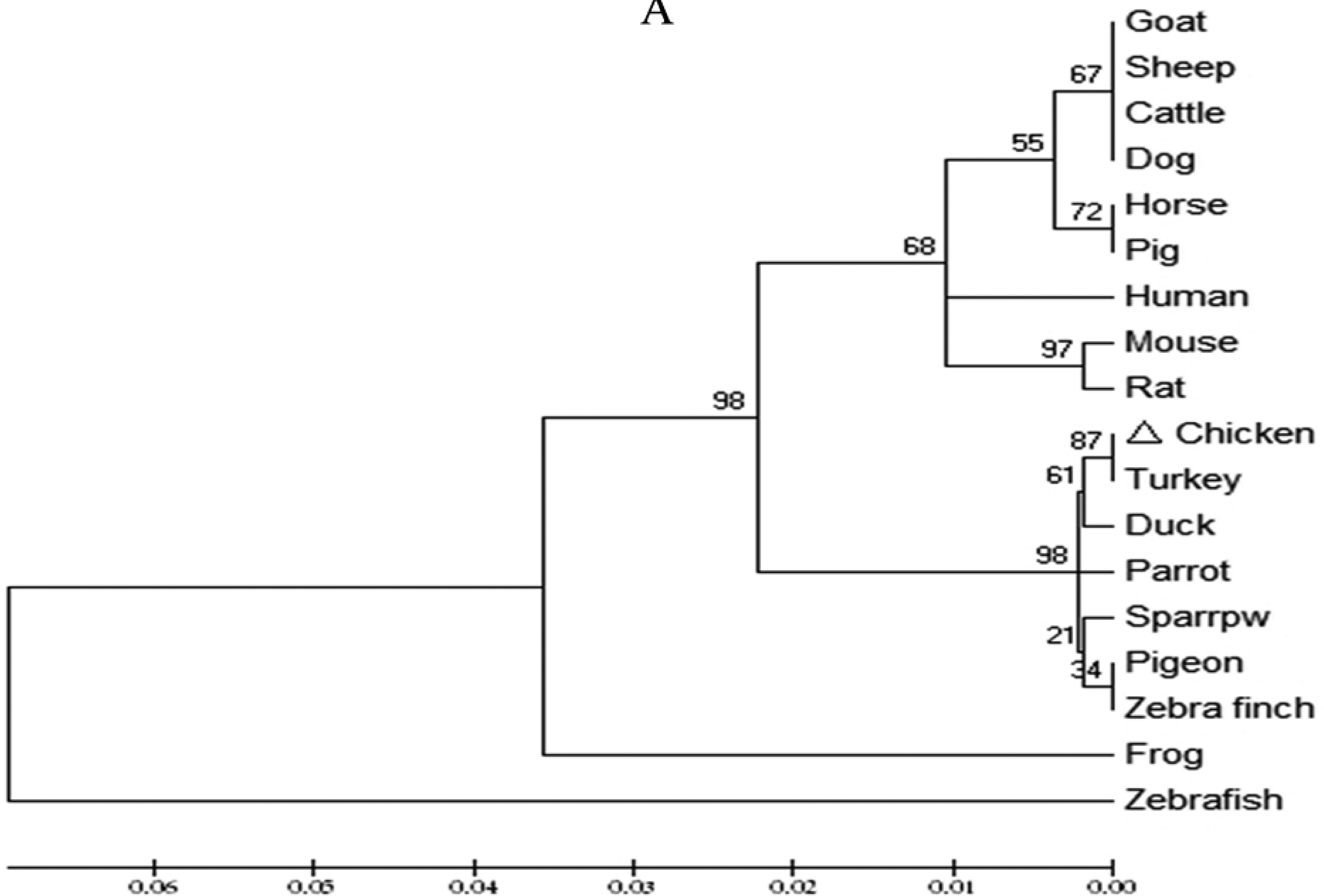

B

Supplement: Figure S1 — (A) Residue substitutions table of homology analysis results. (B) Phylogenetic tree of 18 species based on GNPDA2 homology. Numbers along branch are identities between adjacent species, and the scale line indicates the average genetic distance. Neighbor joining method was used to construct this phylogenetic tree, and the bootstrap value was set as 100. [file peerj-04-2129-s001.pdf]
