# Supplementary material for: Identification, expression and variation of the GNPDA2 gene, and its association with body weight and fatness traits in chicken
Source: PeerJ. 2016 Jun 15;4:e2129. doi: 10.7717/peerj.2129 (PMC4911950; doi:10.7717/peerj.2129)
Supplement: Supplemental Information 1 — Table S1. Nutrient information of high-glucose-fat diet. Table S2. Detailed information of primers and siRNAs of GNPDA2. Table S3. Detailed information of real-time PCR primers. Table S4. Amino acids sequences of GNPDA2 in 18 species. [file peerj-04-2129-s002.docx]

**Table S1. Nutrient information of high-glucose-fat diet.**

| Formula | % by weight | % kcal from |
| --- | --- | --- |
| Protein | 21.2 | 18.3 |
| Carbohydrate | 44.8 | 41.2 |
| Fat | 18.5 | 40.5 |

**Table S2. Detailed information of primers and siRNAs of *GNPDA2*.**

| NO. | Nucleotide sequences (5’→3’） | Size (bp) | Annealing temperature (℃) |
| --- | --- | --- | --- |
| G1 | F:ATGAGGCTTGTCATTCTTGAAGACT | 1062 | 60 |
|  | R: GTGGGTCCACGAGTTTAT |  |  |
| G2 | F: CACCATTGGGCTGCTACA | 875 | 60 |
|  | R: TCGCTTCTCCCGCTGATA |  |  |
| G5’-outer | GGCTGCCCATTCACTTGC | - | 55 |
| G5’-inner | TGGATGATTTCTGGGAAG | - | 60 |
| G3’-outer | ATATGTACATTCACATTCC | - | 55 |
| G3’-inner | TTAGACCTCTGGCTGTTAC | - | 60 |
| QG | F: AGAAGCAGGAGGGATTG  R: CGTTAGCGCCATAGTTG | 168 | 58 |
| QC | F: CAGGATGCAGAAGGAGATC  R: CTGGAAGGTGGACAGGGAG | 127 | 58 |
| SG1 | F: CCAGGTGCTTCCGGATCGTG  R: AATGTTCAACAGTTAATAAG | 1661 | 61 |
| SG2 | F: AGGGAGTACACCATTGGGCTGCTA  R: CCACACCAACCGTTAGCGCCAT | 1262 | 61 |
| SG3 | F: GAATGAATCCTGAATCTCTT  R: ATATACAACCACACATGCTG | 1217 | 58 |
| SG4 | F: TACTTGGCAATCTGTGTAG  R: CCACCACATATTGAAGTTT | 1541 | 60 |
| SG5 | F: GCATATCTTTATTCAACTAA  R: AAACTAAAGAAATGAGACAG | 1461 | 60 |
| SG6 | F: TAGGACCAGAAGGACACT  R: TACAGCAGGACTTGAGAT | 1077 | 61 |
| SG7 | F: AGTGATAGATTATTTCCAAT  R: AAGGATACATTTAAGTTTTAC | 649 | 62 |
| SR1 | S:GGAUGAAUAUGUUGGACUUTT  AS: AAGUCCAACAUAUUCAUCCTT | - | - |
| SR2 | S: GGUGGAAUGUGAUGCAUUUTT  AS: AAAUGCAUCACAUUCCACCTT | - | - |
| SR3 | S: GGAUCGAGUUUGUCUUCAATT  AS: UUGAAGACAAACUCGAUCCTT | - | - |

F and R refer to forward and reverse primer; S and AS refer to sense and antisense siRNA, respectively.

**Table S3. Detailed information of real-time PCR primers.**

| GENE | Nucleotide sequences (5’→3’） | Size  (bp) | Annealing temperature (℃) |
| --- | --- | --- | --- |
| ACC | F: TCGCAGGCATAGCAGGAAAG  R: GTGGACCCCAAAGAAGGAGG | 188 | 58 |
| FAS | F: GGCTGCTGAGGAAGTTGGC  R: AAGTGGCGGGACTGTTTGG | 177 | 60 |
| ATGL | F: ccctgaggatattagatggatg  R: TGCCTGCATGTCCACGT | 286 | 60 |
| HL | F: TTGGCTTACATTTGCTCACC  R: GCTCCTAGGCTGTACCCAATTA | 150 | 60 |
| PPARγ | F: TACTCTCCTGGCTTCTCTC  R: CAAACTCAAACTTGGGCTC | 131 | 58 |
| FTO | F: CCTATGGCTAAACTGGAAG  R: GCTGCCCATTCATTACGC | 167 | 61 |
| PGC1α | F: GACTCAGGTGTCAATGGAAGTG  R: ATCAGAACAAGCCCTGTGGT | 272 | 60 |
| Leptin-R | F: CTTTACATTACCGTGCCTAC  R: AACTGGCGTTGTTATTGC | 176 | 60 |
| PKM | F: AGATTGAGAATCACGAGGG  R: GAAGACTTTTTCAGCAGGG | 113 | 58 |

F and R refer to forward and reverse primer respectively.

**Table S4. Amino acids sequences of *GNPDA2* in 18 species.**

| No. | Species | Latin | NCBI accession number |
| --- | --- | --- | --- |
| 1 | Chicken | Gallus gallus | AFO62196.1 |
| 2 | Duck | Anas platyrhynchos | XP_005015467.1 |
| 3 | Turkey | Meleagris gallopavo | XP_010708526.1 |
| 4 | Parrot | Nestor notabilis | XP_010017946.1 |
| 5 | Sparrpw | Zonotrichia albicollis | XP_005490106.1 |
| 6 | Pigeon | Columba livia | XP_005501288.1 |
| 7 | Zebra finch | Taeniopygia guttata | XP_002197439.2 |
| 8 | Human | Homo sapiens | NP_612208.1 |
| 9 | Mouse | Mus musculus | NP_001033104 |
| 10 | Rat | Rattus norvegicus | NP_001099475.1 |
| 11 | Dog | Canis familiaris | XP_849417.1 |
| 12 | Pig | Sus scrofa | XP_003128996.1 |
| 13 | Cattle | Bos taurus | NP_001068824.1 |
| 14 | Sheep | Ovis aries | XP_004009851.1 |
| 15 | Goat | Capra hircus | XP_005681624.1 |
| 16 | Horse | Equus caballus | XP_001917218.1 |
| 17 | Frog | Xenopus (Silurana) tropicalis | NP_001096324.1 |
| 18 | Zebrafish | Danio rerio | XP_684147.2 |
